# Loss of HER2 Positivity after Trastuzumab in HER2-Positive Gastric Cancer: Is Change in HER2 Status Significantly Frequent?

**DOI:** 10.1155/2015/132030

**Published:** 2015-03-29

**Authors:** Yu Ishimine, Akira Goto, Yoshito Watanabe, Hidetaka Yajima, Suguru Nakagaki, Takashi Yabana, Takeya Adachi, Yoshihiro Kondo, Kiyoshi Kasai

**Affiliations:** ^1^Department of Gastroenterology, Otaru General Hospital, 1-2-1 Wakamatu, Otaru 047-8550, Japan; ^2^Department of Surgery, Otaru General Hospital, 1-2-1 Wakamatu, Otaru 047-8550, Japan; ^3^Department of Gastroenterology, Rheumatology, and Clinical Immunology, Sapporo Medical University, S1W17, Chuo-ku, Sapporo 060-8556, Japan; ^4^Department of Laboratory Medicine, Otaru General Hospital, 1-2-1 Wakamatu, Otaru 047-8550, Japan

## Abstract

Trastuzumab has recently been introduced as a treatment for HER2-positive metastatic and/or unresectable gastric cancer (MUGC); however, compared with breast cancer, some issues concerning HER2 and trastuzumab therapy for gastric cancer remain unclear. A 74-year-old woman received trastuzumab-containing chemotherapy for HER2-positive MUGC. She had a marked response to 8 months of chemotherapy, and gastrectomy and hepatic metastasectomy with curative intent were performed. The resected specimen showed complete loss of HER2 positivity in the residual tumor. For MUGC, a change in HER2 status during the course of the disease with or without chemotherapy has rarely been reported. However, in breast cancer, a significant frequency of change in HER2 status during the course of disease has been reported, and reevaluation of HER2 positivity in metastatic/recurrent sites is recommended. The choice of trastuzumab for MUGC is currently based on the HER2 status of the primary tumor at the time of initial diagnosis, without reassessment of HER2 status during the course of disease and/or in metastatic/recurrent sites, on the assumption that HER2 status is stable. However, our case casts doubt on the stability of HER2 in gastric cancer.

## 1. Introduction

The primary treatment for patients with metastatic and/or unresectable gastric cancer (MUGC) is systematic chemotherapy. Although the combination of 5-fluorouracil and cisplatin has been the standard regimen for the treatment of MUGC, new cytotoxic agents such as S-1, capecitabine, paclitaxel, docetaxel, oxaliplatin, irinotecan, and their combinations have proven to be more effective and better tolerated, resulting in the improvement of median survival up to 13 months [1–3]. In addition, recent research has shown that adding trastuzumab, a humanized monoclonal antibody targeting the human epidermal growth factor receptor 2 (HER2) protein, to chemotherapy (fluoropyrimidines and cisplatin) for patients with MUGC with HER2 overexpression and/or gene amplification (the so-called HER2-positive gastric cancer) has a significant superiority to chemotherapy alone in terms of overall survival (median survival 13.8 months with trastuzumab versus 11.1 months with chemotherapy alone) [4].

We report a case in which chemotherapy containing trastuzumab made it possible to perform curative gastrectomy in a patient with HER2-positive MUGC at initial diagnosis; this case is noteworthy in that HER2 positivity in resected specimens of the residual tumor was completely lost. Loss of HER2 positivity after treatment with trastuzumab for HER2-positive MUGC has rarely been reported. HER2 status is an essential biomarker for the effectiveness of trastuzumab, and consequently, knowledge of HER2 stability during the course of disease is critical for determining the introduction, the continued use, or elimination of trastuzumab. We discuss the stability of HER2 status in gastric cancer and provide a literature review of this important topic.

## 2. Case Report

**Figure 1:**
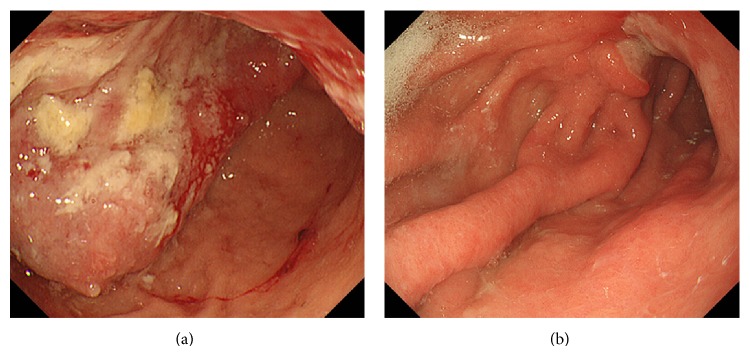
Endoscopic findings. (a) Gastroscopy at presentation revealed an ulcerative mass with raised margins along the lesser curvature extending from the lower body of the stomach to the antrum. (b) After completion of the 11th course of chemotherapy (capecitabine + CDDP + trastuzumab), the gastric tumor is decreased remarkably.

**Figure 2:**
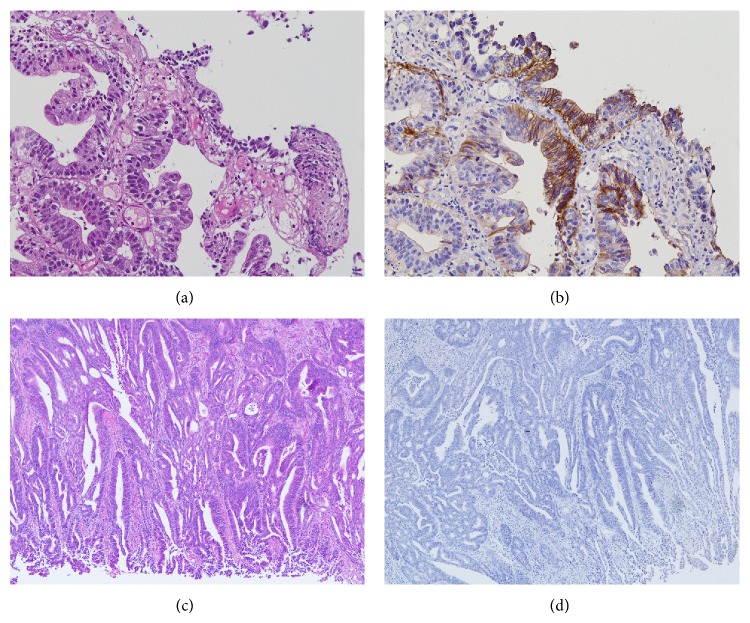
Microscopic findings of gastric cancer. (a) and (b) show biopsy specimens at presentation. (a) Histological findings show a well-differentiated tubular adenocarcinoma (hematoxylin and eosin staining, original magnification ×40). (b) Immunohistological findings of HER2 immunostaining show strong basolateral membrane reactivity with heterogeneity. The gastric cancer is scored as 3+ by HER2 immunohistochemistry scoring criteria (original magnification ×40). (c) and (d) show the resected specimen after chemotherapy including trastuzumab. (c) Histological findings show a moderately differentiated tubular adenocarcinoma invading into the subserosa (hematoxylin and eosin staining, original magnification ×40). (d) Immunohistological findings of HER2 immunostaining showed no membranous reactivity in any tumor cells (original magnification ×40).

**Figure 3:**
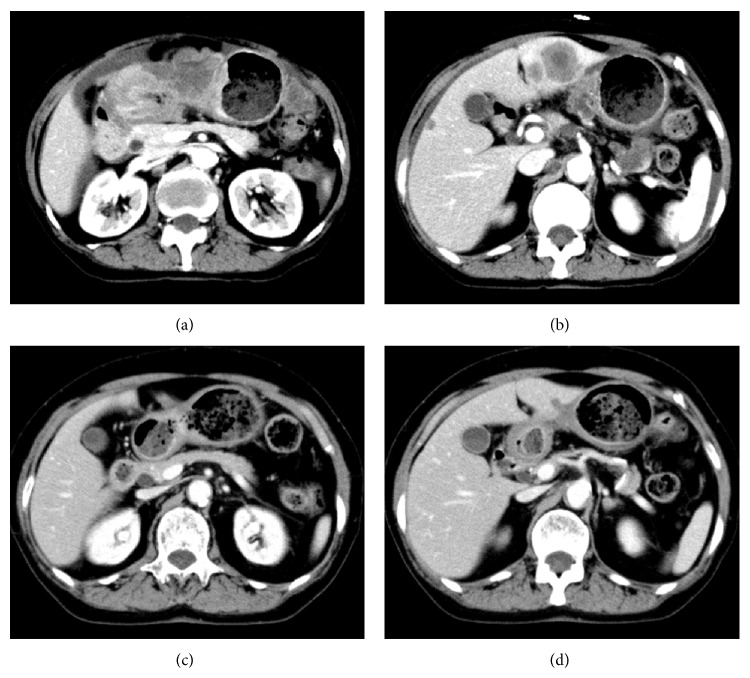
Contrast-enhanced computed tomography (CT) findings. (a) and (b) show the CT at presentation. (a) The wall of the lower part of the stomach is markedly thickened, and the density of the surrounding fat tissue is increased, forming an 80 × 45 mm mass. (b) Two nodules are detected in the lateral segment of the liver, and these are directly contiguous from the gastric mass. (c) and (d) show the CT findings after completion of the 11th course of chemotherapy (capecitabine + CDDP + trastuzumab). (c) The gastric mass is markedly decreased. (d) Only a small nodule in the lateral segment of the liver is detected.

A 74-year-old woman with the chief complaint of palpable masses in her abdomen presented to our hospital in May 2013. Physical examination revealed one 50-mm mass in the upper central abdomen and three 20–30-mm palpable masses in the other parts of her abdomen. Esophagogastroduodenoscopy (EGD) revealed an ulcerative mass with raised margins along the lesser curvature extending from the lower body of the stomach to the antrum (Figure 1(a)). Biopsy of the lesion revealed a well-differentiated tubular adenocarcinoma (Figure 2(a)), and HER2 immunohistochemistry (IHC) scoring of the tumor in the biopsy specimen was 3+, indicating HER2-positivity (Figure 2(b)). Contrast-enhanced computed tomography (CT) demonstrated marked wall thickening of the lower part of the stomach with irregular outer borders and an increased density of the surrounding fat tissue, forming a mass that was 80 × 45 mm (Figure 3(a)). Several nodules in the liver were detected. Two of these nodules in the lateral segment of the liver were directly contiguous from the gastric mass and were considered direct invasion of the gastric cancer (Figure 3(b)). Invasion into the pancreas was suggested by obliteration of the fat plane between the gastric mass and the pancreas. CT also revealed multiple nodules in the peritoneal cavity, a few masses in the abdominal wall, and ascites. Many perigastric lymph nodes and several para-aortic lymph nodes were swollen.

With this information, this patient's gastric cancer was classified as stage IV (cT4bN3aM1) according to the 7th TNM classification of the American Joint Committee on Cancer/Union for International Cancer Control (AJCC/UICC) staging system for gastric cancer. She was treated with 3-week courses of capecitabine (2000 mg/m^2^/day) orally on days 1–14, cisplatin (80 mg/m^2^) intravenously on day 1, and trastuzumab (8 mg/kg loading dose followed by 6 mg/kg) intravenously on day 1. From the 3rd course, the doses of capecitabine and cisplatin were reduced to 1500 mg/m^2^/day and 60 mg/m^2^, respectively, due to nonhematologic toxicity (nausea, anorexia, and renal impairment). After completion of the 4th course of chemotherapy, at 3 months after diagnosis, a 76% decrease in measureable tumor burden compared to baseline according to RECIST 1.1 criteria was observed. From the 6th course, cisplatin was discontinued due to renal impairment, but treatment with capecitabine and trastuzumab continued. After completion of the 11th course, at 8 months after diagnosis, EGD showed a marked reduction of the gastric tumor (Figure 1(b)), and contrast-enhanced CT (Figures 3(c) and 3(d)) showed a 90% reduction in measureable tumor burden. No metastases were detected in the liver except for one small nodule in the lateral segment (Figure 3(d)). Ascites and metastases in the peritoneal cavity, abdominal wall, and para-aortic lymph nodes were completely resolved.

Given this response to the treatment, complete resection of the residual tumor was deemed possible, and 9 months after the diagnosis, distal gastrectomy with D2 lymphadenectomy and hepatic metastasectomy of the small nodule in the lateral segment were performed. Operative findings showed no macroscopic peritoneal metastasis and the absence of peritoneal lavage cytology. Macroscopic complete resection was possible. Pathologic findings of the 36 × 25 mm mass from the resected stomach revealed a moderately differentiated adenocarcinoma with subserosal invasion (Figure 2(c)) and hyalinization and fibrosis adjacent to the tumor in the lower third of the gastric wall. Eight regional lymph nodes were positive for tumor cells. Specimens of the nodule from the resected liver revealed only hyalinization and fibrosis without tumor cells. HER2 by IHC was completely negative in the residual tumor from the resected stomach (Figure 2(d)) and lymph nodes. HER2 also was negative by fluorescence in situ hybridization (FISH). The patient received adjuvant chemotherapy comprising capecitabine and trastuzumab for 6 months, and at the completion of adjuvant chemotherapy, the patient had no signs of recurrence.

## 3. Discussion

Approximately 15–20% of invasive breast cancers have HER2 overexpression and/or gene amplification [5, 6]. HER2 has been used as a molecular target for breast cancer for many years. Trastuzumab was initially approved for the treatment of patients with HER2-positive metastatic breast cancer [7]. Trastuzumab is now the standard treatment in adjuvant therapy for HER2-positive early-stage breast cancer [8] and is also recommended as part of neoadjuvant chemotherapy (NAC) [9].

In breast cancer, discordance in HER2 status between the primary tumor and the metastasis/recurrence and HER2 stability during the course of disease has been evaluated in several reports. The discordance rate of HER2 status in breast cancer has been reported to be 3–16% [10–13], with 12–19% of HER2-positive tumors in the primary site converting to HER2-negative status at the sites of metastasis/recurrence [11, 12] and 7–10% of HER2-negative tumors converting to HER2-positive status [11, 12, 14]. Conversion of the HER2 status from positive to negative may be more frequent than conversion from negative to positive. The cause of discordance of HER2 status has been attributed to changes in the molecular profile during the course of tumor progression, the differing effects of prior treatment on clonal subsets, heterogeneity of HER2 expression within the tumor, and technical errors in tissue processing and evaluation.

Several previous reports have evaluated how chemotherapy with or without anti-HER2 agents, including trastuzumab, influences HER2 expression. The discordance rate of HER2 status before and after NAC has been reported to be 0–9.5% [15–17]. In HER2-positive breast cancer, loss of HER2 positivity after NAC has been reported to be 8–32% [15, 17–19], and in HER2-negative breast cancer, gain of HER2 positivity after NAC has been reported in 0–5% of cases [15, 17]. Niikura et al. reported that the conversion rate from positive in the primary tumors to negative in the sites of metastases was significantly higher in patients with prior chemotherapy than in patients without prior chemotherapy (17% versus 10%) [20]. These previous reports have suggested that chemotherapy can be associated with a change of HER2 status in breast cancer.

In addition to studying the association between HER2 status and traditional chemotherapeutic agents, several reports have analyzed the association between change in HER2 status and trastuzumab therapy. A loss of HER2 positivity in patients treated with trastuzumab therapy has been reported in 14.7–37% of HER2-positive breast cancers [14, 18–21]. Nakamura et al. reported that, in HER2-positive primary breast cancer, the rate of HER2 loss in the site of recurrence was significantly higher in patients treated with trastuzumab than in patients treated without trastuzumab (37% versus 6%) [11]. However, others have reported that trastuzumab therapy was not significantly associated with loss of HER2 positivity in HER2-positive primary breast cancer (13.2% with trastuzumab versus 17.9% without trastuzumab and 20% with trastuzumab versus 26% without trastuzumab) [14, 20]. In fact, some research has shown that treatment with trastuzumab is associated with a lower rate of HER2 status conversion. Guarneri et al. reported that in HER2-positive breast cancer the rate of loss of HER2 after NAC with trastuzumab was significantly lower than the rate of conversion after treatment with NAC without trastuzumab (14.7% versus 40%) [19]. It is evident that available results on the association between loss of HER2 status and trastuzumab therapy are conflicting. These conflicting results imply that loss of HER2 in HER2-positive breast cancer after trastuzumab therapy might not simply be caused by survival of trastuzumab-resistant clones and elimination of HER2-positive clones.

HER2 overexpression and/or amplification occurs in 7–34% of gastric cancer cases [22, 23]. In gastric cancer, the rate of HER2 status discordance between primary tumors and simultaneous metastasis in the liver or lymph nodes has been reported to be 0–12.5% [24–26]. However, HER2 stability during disease progression with or without chemotherapy has rarely been reported. In fact, our literature review identified only one case report similar to ours in which HER2 positivity in the resected specimen from curative surgery was lost after 12 courses of capecitabine + cisplatin + trastuzumab in a patient with HER2-positive MUGC [27]. However, the actual frequency of HER2 loss in HER2-positive MUGC remains unknown, because in MGC, reevaluation of HER2 status in primary sites and/or sites of metastases/recurrence during the course of disease is not typically performed.

When analyzing loss of HER2 in a sample obtained after treatment, we must consider the possibility of a false-negative result for HER2 expression on immunohistochemistry. A sample taken by biopsy is only part of a tumor, and a resected sample might be one of many tumors within the body; therefore, loss of HER2 might be a false-negative due to heterogeneity within the tumor or discordance between the sites of tumors. In fact, it is known that the heterogeneity of HER2 expression within gastric tumors is much more frequent than in breast cancer [22, 23]. Therefore, heterogeneity might account for a small fraction of HER2 conversion. Resolution of this issue is quite challenging, because it is difficult in practice to obtain adequate specimens to overcome the heterogeneity of HER2 within a given tumor and to evaluate the HER2 status of every tumor within the entire body. However, we believe that our particular case was not due to a false-negative result of HER2, because we carefully evaluated HER2 status in the entire residual tumor of the resected stomach and lymph nodes. Although previous studies have demonstrated that 0–10% of cases negative for HER2 by IHC were positive by FISH [24, 25, 28], we confirmed negativity of HER2 in this case by FISH as well.

Current practice guideline for the treatment of breast cancer recommended that biomarkers, including HER2 status and hormone receptors, should be evaluated not only for the primary tumor but also for the initial metastatic or recurrence tumor tissues, especially if the first determination of HER2 status is negative, in order not to lose the chance of anti-HER2 therapy [29]. Currently, the decision to use trastuzumab to treat patients with MUGC is based on the HER2 status in the primary tumor at the time of initial diagnosis without reassessment of HER2 status in primary sites and/or metastases/recurrence during the course of disease, on the assumption that HER2 status is stable. However, just as it is known that HER2 is not necessarily stable in breast cancer, our case casts some doubt on the notion of HER2 stability in gastric cancer.

Although the use of trastuzumab has been shown to be highly effective in treating HER2-positive MUCG, several issues regarding HER2 stability and trastuzumab therapy for gastric cancer remain to be elucidated. First, clinicians need to know how often HER2 status converts with tumor progression, metastasis, or treatment. If a change in HER2 status is found to be significantly frequent in gastric cancer, management strategy recommendations similar to those of breast cancer should be introduced for gastric cancer, although it will be important to consider that biopsy from metastases/recurrence or metastasectomy is invasive and burdensome for patients. If the factors that contribute to HER2 change are identified, only those patients who have such factors, rather than all patients with MUGC, would need to submit to biopsy from metastases/recurrence or metastasectomy. Second, clinicians need to evaluate whether loss of HER2 positivity in specimens obtained during the disease course in HER2-positive gastric cancer always indicates resistance to trastuzumab. As we have mentioned, HER2 status taken from samples may not always represent all tumors in the body. In the present case, although macroscopic complete resection was possible, there was still a sufficient possibility of remaining micrometastases. We decided to treat the patient with adjuvant therapy comprised of capecitabine and trastuzumab after the patient gave her informed consent. Therapy comprised of capecitabine and trastuzumab was chosen because her tumors were not resistant to these medications until resection, and trastuzumab has a significant benefit in tumor cell eradication in the event that the remaining micrometastases retain HER2-positivity. Third, clinicians need to know if the loss of HER2 positivity has a significant prognostic impact on MUGC, because some studies have shown that loss of HER2 positivity in HER2-positive breast cancer was associated with a significantly worse prognosis compared to patients with tumors that maintained HER2 expression [12, 18, 20, 21].

Even if a marked response to chemotherapy allows the possibility of curative resection in MUGC, currently, the consistent standard treatment is to continue chemotherapy. However, the survival rate in MUGC treated by chemotherapy alone is obviously insufficient. Surgical resection with curative intent after a response to chemotherapy for MUGC is called adjuvant or salvage surgery. The benefit of such surgery was recently evaluated, and a favorable prognosis was suggested, with a 3-year survival rate of 46–55% and a median survival of 29–43 months [30–32], although these studies were small and retrospective in nature. In carefully selected patients, adjuvant surgery might become the treatment of choice. To support the efficacy of adjuvant surgery, a randomized controlled study is warranted.

In conclusion, we report the case of a patient with HER2-positive MUGC at initial diagnosis who received chemotherapy including trastuzumab; following chemotherapy and surgery in this patient, a resected specimen of the stomach showed loss of HER2-positivity in the residual tumor, suggesting that HER2 status is not always stable. Treatment with trastuzumab is very effective and less toxic than traditional cytotoxic drugs. If HER2 positivity is gained during the course of disease, it is an easy judgment for clinicians to add trastuzumab to chemotherapy, thus allowing patients to benefit from trastuzumab therapy. However, when cases convert from positive HER2 status to negative HER2 status, clinicians remain uncertain as to whether the change in status is truly due to a biologic change rather than a false-negative result. Therefore, it is a more difficult and less confident judgment to remove trastuzumab from chemotherapy, even though its removal may result in avoiding unnecessary treatment. Further studies concerning HER2 status in gastric cancer are needed in order to appropriately use trastuzumab for these patients.
